# Battery‐Free, Stretchable, and Autonomous Smart Packaging

**DOI:** 10.1002/advs.202417539

**Published:** 2025-05-12

**Authors:** Ali Douaki, Mukhtar Ahmed, Edoardo Longo, Giulia Windisch, Raheel Riaz, Sarwar Inam, Thi Nga Tran, Evie L. Papadopoulou, Athanassia Athanassiou, Emanuele Boselli, Luisa Petti, Paolo Lugli

**Affiliations:** ^1^ Sensing Technologies Laboratory (STL) Faculty of Engineering Free University of Bozen‐Bolzano Piazza Università 5 Bozen 39100 Italy; ^2^ Faculty of Agricultural Environmental and Food Sciences Free University of Bozen‐Bolzano Piazza Università 5 Bozen 39100 Italy; ^3^ Smart Materials Group Istituto italiano di Tecnologia via Morego 30 Genova 16163 Italy; ^4^ Optoelectronics Research Line Istituto italiano di Tecnologia via Morego 30 Genova 16163 Italy; ^5^ Dip. di Scienze e Metodi dell'Ingegneria Università di Modena e Reggio Emilia via Amendola 2 Reggio Emilia 42122 Italy; ^6^ ABB Corporate Technology Center Krakow 31‐038 Poland; ^7^ Bedimensional SPA Lungotorrente Secca, 30R Genova 16163 Italy

**Keywords:** PNIPAM, intelligent packaging, NFC antenna, smart materials

## Abstract

In the food industry, innovative packaging solutions are increasingly important for reducing food waste and contributing to global sustainability efforts. However, current food packaging is generally passive and unable to adapt to changes in the food environment in real time. To address this, a battery‐less and autonomous smart packaging system is developed that wirelessly powers closed‐loop sensing and release of active compounds. This system integrates a gas sensor for real‐time food monitoring, a Near‐Field Communication (NFC) antenna, and a controlled release of active compounds to prevent quality deterioration in the complex food environment. The ability of the developed smart packaging system is demonstrated, to continuously monitor the freshness of fish products and to trigger the release of active compounds when the food starts to spoil. The system is able to extend the shelf‐life of the food product up to 14 days, due to the controlled release of antioxidant and antibacterial compounds. The system can pave the way toward an Internet of Things solution that addresses protection, active prevention of food spoilage, and sustainability, facing all the current challenges of the food packaging industry.

## Graphical abstract

This article reports the development of a battery‐free, autonomous smart packaging system that integrates real‐time food monitoring through a gas sensor, a Near‐Field Communication (NFC) antenna, and a controlled release of active compounds. The system effectively extends food shelf‐life by up to 14 days, demonstrating its potential to preserve food quality and prevent spoilage.

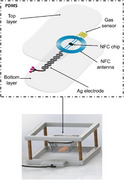

## 1 Introduction

The need for sustainable and safe packaging solutions continues to grow, driven by increasing consumer awareness and environmental concerns.^[^ ^1^ ^]^ This has become even more crucial given the persistent global issues of food waste and spoilage. According to estimates, roughly one‐third of all food produced is wasted or lost annually, leading to significant economic, social, and environmental impacts.^[^ ^2^ ^]^ In addition to the financial cost of wasted food, which can amount to billions of dollars annually, the production and transportation of food that is ultimately discarded also have environmental consequences, including greenhouse gas emissions and resource depletion.^[^ ^3^ ^]^ The main cause of food spoilage is oxidation due to contact with air over a prolonged period and, second, the uncontrolled development of micro‐organisms.^[^ ^4^, ^5^ ^]^ To prolong the shelf life of food, considerable efforts are being undertaken to delay the occurrence and extent of these processes using advanced food packaging. It is therefore crucial to develop sustainable and innovative packaging solutions that can preserve the quality and extend the shelf life of products, ultimately reducing waste and benefiting both consumers and the environment.

Advancements in active and intelligent packaging technologies have been undertaken to reduce food waste, primarily through shelf‐life extension or by monitoring food product quality. Active packaging incorporates components like oxygen scavengers,^[^ ^6^, ^7^ ^]^ moisture absorbers,^[^ ^6^, ^8^ ^]^ and antimicrobial agents,^[^ ^9^, ^10^, ^11^, ^12^, ^13^ ^]^ to enhance product quality and prolong shelf life.^[^ ^14^, ^15^ ^]^ On the other hand, intelligent packaging employs sensors, indicators, and RFID tags for real‐time monitoring of product conditions.^[^ ^16^, ^17^, ^18^, ^19^, ^20^, ^21^, ^22^, ^23^ ^]^ However, both technologies have limitations that render them ineffective, especially when applied independently.

The existing working mechanism of innovative packaging for extending food shelf life relies on oxygen absorption, bio‐active compound release, and volatile organic compound (VOC) concentration monitoring, which are only moderately effective.^[^ ^24^ ^]^ This is because these technologies generally exhibit a passive nature, lacking the ability to actively adapt to fluctuations in the food package environment.^[^ ^24^ ^]^ For instance, active packaging, while autonomously functional, is incapable of responding to changing environmental variables, and the continuous release of bio‐active compounds into the packaging space, governed by Fick's law of diffusion, can compromise the food's freshness.^[^ ^25^, ^26^ ^]^ This may even lead to microbial resistance in food products, posing potential public health risks and economic losses.^[^ ^26^, ^27^ ^]^ Efforts to synchronize the release rate of these compounds with the spoilage rate of food products have proven challenging and largely unsuccessful.^[^ ^28^ ^]^ Intelligent packaging, on the other hand, depends on external sensors and indicators to provide real‐time data on product conditions. However, this technology falls short in applications requiring a longer shelf life, as it does not actively interact with the product environment, hence, a quest to find new smart packaging solutions has emerged. Moreover, so far, multiple separate devices have been reported in the literature such as gas sensors,^[^ ^29^ ^]^ NFC antennas, and controlled release mechanisms.^[^ ^9^ ^]^ A smart packaging solution that combines the strengths of active and intelligent packaging, while addressing their limitations, has the potential to significantly improve food safety, extend shelf life, and reduce waste. This would benefit consumers, manufacturers, and the environment.

To achieve an improved food packaging system, it should meet the following requirements: i) controlled release of antioxidants with zero leakage in the off state, ii) integration of sensing and autonomous, closed‐loop, food freshness management, iii) wirelessly powered to avoid having batteries in direct contact with food, iv) stability under harsh environment. Such a system should also fulfill the 4S (scalable, stretchable, self‐powered, and sensing).

This study aims to advance the existing literature by developing a comprehensive smart packaging system through the integration of individual devices into a complete battery‐free, stretchable, and autonomous smart packaging, designed to extend the shelf life of food products. The system combines the benefits of both active and intelligent packaging technologies, with the intelligent packaging component monitoring the freshness of the food product and activating the release of the antioxidants from the active packaging component only when the food starts to deteriorate. To enable autonomous operation, the two components are linked via an NFC antenna that wirelessly powers the system and triggers the release of the antioxidants. Each component of the smart packaging system was characterized separately and then as a complete system. To validate the efficacy of the smart packaging system, we employed gas chromatography/ mass spectrometry (GC/MS) to measure the release of cinnamaldehyde (CA) and eugenol (EG) inside the packaging space. Our results show that food waste can be reduced by using an innovative smart packaging system. Furthermore, the system can also be integrated with IoT to improve supply chain management.

## 2 Results and Discussion

### 2.1 Wireless and Battery‐Free Smart Packaging System

**Figure 1:**
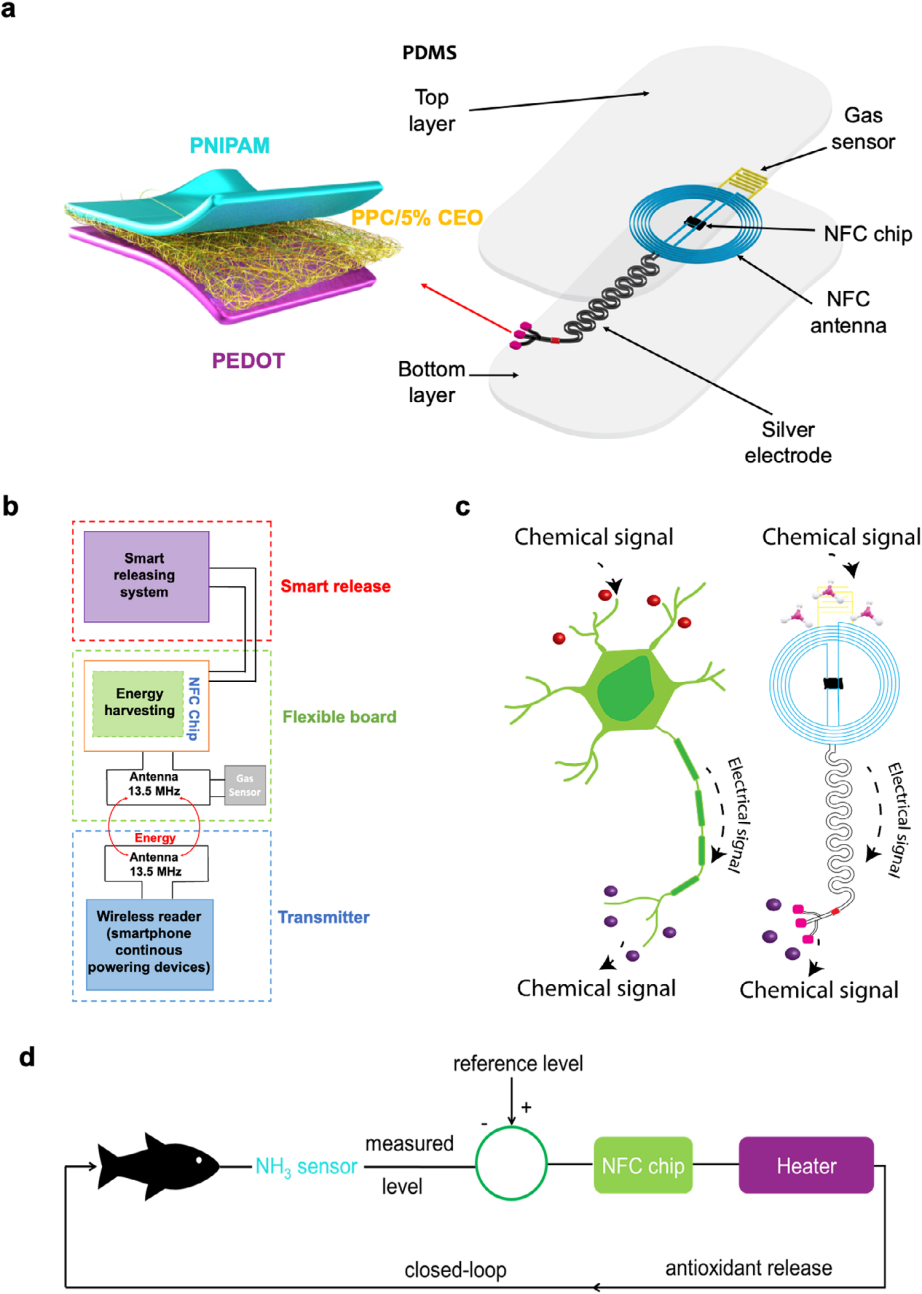
Device concept. a) Schematic illustrating the exploded view of the complete hybrid, battery‐free system. b) Different parts of the smart packaging. c) Working mechanism of the smart packaging. d) Image illustrating the closed‐loop system.

The developed smart packaging utilizes a gas sensor, an NFC antenna, and smart materials to control the release of active compounds. **Figure** **1**a shows a schematic illustration of the smart packaging, highlighting the three main components, i.e., the single‐walled carbon nanotube (SWCNT) gas sensor, the wireless platform, and the controlled release system. The wireless platform consists of i) a power harvesting coil that operates by magnetic inductive coupling at a resonance frequency of 13.56 MHz, to power the system, ii) a near‐field communication (NFC) system on chip (M24LR16E) that supports wireless communication, iii) a SWCNT gas sensor that serves as a food spoilage detector and the switch to trigger the release of the antimicrobial agent namely cinnamon essential oil (CEO), and iv) electrospun polypropylene carbonate (PPC) mat containing active compound that releases under a thermal stimulation provided by the heater at the bottom of the mat “Poly(3,4‐ethylenedioxythiophene)‐poly(styrenesulfonate) (PEDOT:PSS)” (Figure 1b).

Figure 1c elucidates the working principle of smart packaging, which draws inspiration from the functional mechanism of a neuron. Like a neuron's response to a chemical stimulus, the smart packaging transduces chemical signals into electrical impulses. In this case, food spoilage coincides with the formation of ammonia (NH_3_) used here as an indicator—being released into the headspace of the packaging. Ammonia interacts with the SWCNT gas sensor, causing an increase in its resistance. This resistance change affects the connected antenna's resonance frequency, enhancing the NFC antenna's gain and, consequently, the voltage harvested by the NFC antenna. This harvested voltage is then supplied to the PEDOT:PSS layer to elevate the mat's temperature above the lower critical solution temperature (LCST) of 32 °C for Poly(N‐isopropylacrylamide) (PNIPAM). The latter is used to control active compound release. At room temperature, PNIPAM remains in a swelled state, inhibiting the release of the active compounds (Note S1. in Supporting Information). However, surpassing the LCST causes PNIPAM to collapse, triggering the discharge of the active compound from the fibers.^[^ ^9^ ^]^ The developed packaging works as a closed‐loop system that continuously monitors and adapts the conditions of the food packaging headspace, and thus preserves in a dynamic way the food freshness (Figure 1d).

### 2.2 Characterization of the Gas Sensor

**Figure 2:**
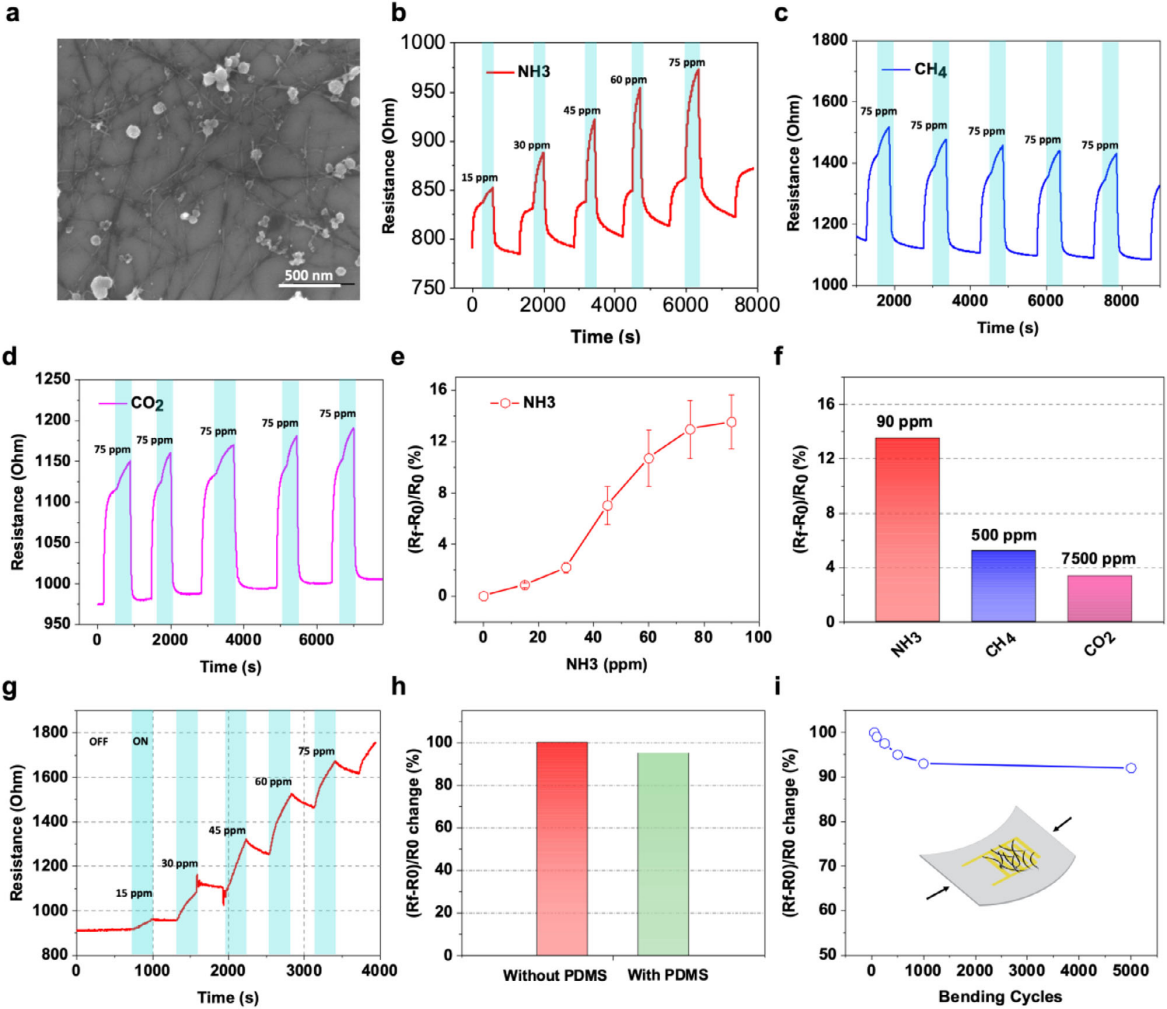
a) Scanning electron microscopy (SEM) image of the spray‐coated SWCNTs on a silicon substrate. b) Response of CNTs gas sensor toward ammonia for concentrations ranging from 15 to 75 ppm with a step of 15 ppm, the blue zones represent the exposure zone followed by a recovery step via external heating. c) Response of CNTs gas sensor toward different concentrations of methane. d) Response of CNTs gas sensor toward different concentrations of carbon dioxide, e) Calibration curve of CNTs gas sensor toward ammonia. f) Selectivity test of CNTs gas sensor in the presence of 90 ppm ammonia, 500 ppm methane, and 7500 ppm carbon dioxide. g) Resistance change of the gas sensor in the presence of different ammonia concentrations ranging from 15 to 90 ppm with a step of 15 ppm without a recovery step. h) The response of the CNTs gas sensor toward ammonia without and with PDMS for 90 ppm of NH_3_ to investigate the effect of PDMS on the response of the gas sensor. i) The effect of the mechanical stress “bending” on the performance of the CNTs gas sensor on PDMS substrate “in the presence of 90 ppm NH_3_”.

Previous studies have established ammonia (NH_3_) as a robust biomarker for detecting fish spoilage.^[^ ^30^ ^]^ This is because NH_3_ is produced by bacteria during protein spoilage and accumulates in the headspace of spoiled fish packaging. Consequently, NH_3_ detection offers a non‐invasive alternative to conventional methods and the capability for rapid and real‐time monitoring.^[^ ^31^, ^32^, ^33^, ^34^ ^]^ In this work, we have developed the SWCNT gas sensor to assess the freshness of fish products by quantifying NH_3_ levels in the headspace of the food packaging. This section focuses on the fabrication and characterization of this gas sensor. **Figure** **2**a displays a scanning electron microscope (SEM) image of a 25‐layer spray‐deposited SWCNT film. This film was optimized in terms of the number of sprayed layers to attain an initial resistance of 1 kOhm (explained in section 5). The image reveals a uniform, homogeneous, and sparsely distributed network of SWCNTs. Figure 2b–d illustrates the SWCNT gas sensor's resistance variation over time following exposure to different NH_3_, CH_4_, and CO_2_ gas concentrations. Additionally, Figure 2e exhibits the gas sensor's linear response to NH_3_‐increasing concentrations ranging from 15 ppm to 90 ppm, followed by sensor saturation. Notably, the SWCNT gas sensor demonstrated heightened sensitivity to NH_3_ compared to CH_4_ and CO_2_. The resistance increase is attributed to the SWCNT gas sensor's mechanism, extensively documented in the literature.^[^ ^35^, ^36^, ^37^, ^38^ ^]^ The smart packaging developed in this research utilizes the resistance increase in the gas sensor to initiate release mechanisms. Therefore, gas selectivity is a crucial aspect for ensuring reliable and precise detection, as lower specificity for gases could lead to false system activation. Consequently, the selectivity of the gas sensors against NH_3_, CH_4_, and CO_2_ was thoroughly examined. The results demonstrated a significant 13% response increase in the gas sensor when exposed to 90 ppm of NH_3_. In contrast, exposure to 500 ppm of CH_4_ and 7500 ppm of CO_2_ resulted in only a 5% and 3.5% response increase, respectively, as depicted in Figure 2f. Despite higher concentrations of CH_4_ and CO_2_, the sensor's response to NH_3_ was more pronounced, indicating the sensor's favorable selectivity toward NH_3_. Afterward, the change of the SWCNT gas sensor in the presence of NH_3_ without recovering the gas sensor was investigated and reported in Figure 2g, as we can observe the increase in NH_3_ concertation led to an increase in the resistance of the gas sensor from 900 to 1800 Ohm in a concentration of NH_3_ ranging from 15 ppm to 90 ppm. This increase in resistance will later be employed to trigger the release of the active compounds. Furthermore, the passive recovery of the gas sensor over time below the threshold resistance will act as a switch‐off to stop the release of the active compound.

Moreover, the humidity notably influences the resistance of SWCNTs as has been previously reported.^[^ ^39^ ^]^ Given that humidity levels inside food packaging often hover at 90% (Figure S2, Supporting Information), this becomes a significant factor in gas sensor design for such applications. To address humidity interference, a thin polydimethylsiloxane (PDMS) layer was applied to passivate the SWCNT gas sensor. PDMS, being porous yet hydrophobic, permits gas molecules to permeate while barring water molecules.^[^ ^40^ ^]^ Therefore, the influence of PDMS passivation on the sensor's sensitivity was assessed, as shown in Figure 2h. The figure displays the SWCNT gas sensor's response to 90 ppm of NH_3_. The passivated sensor showed a mere 5% decrease in response, likely due to the NH_3_ molecules' ability to penetrate the PDMS layer easily. Mechanical stability of the gas sensor is another important parameter, hence, the effect of mechanical stress in terms of bending was investigated. As shown in Figure 2i, the gas sensor showed good stability even after 5000 bending cycles where the gas sensor lost ≈5% of the original sensitivity.

### 2.3 NFC Antenna in Smart Food Packaging

**Figure 3:**
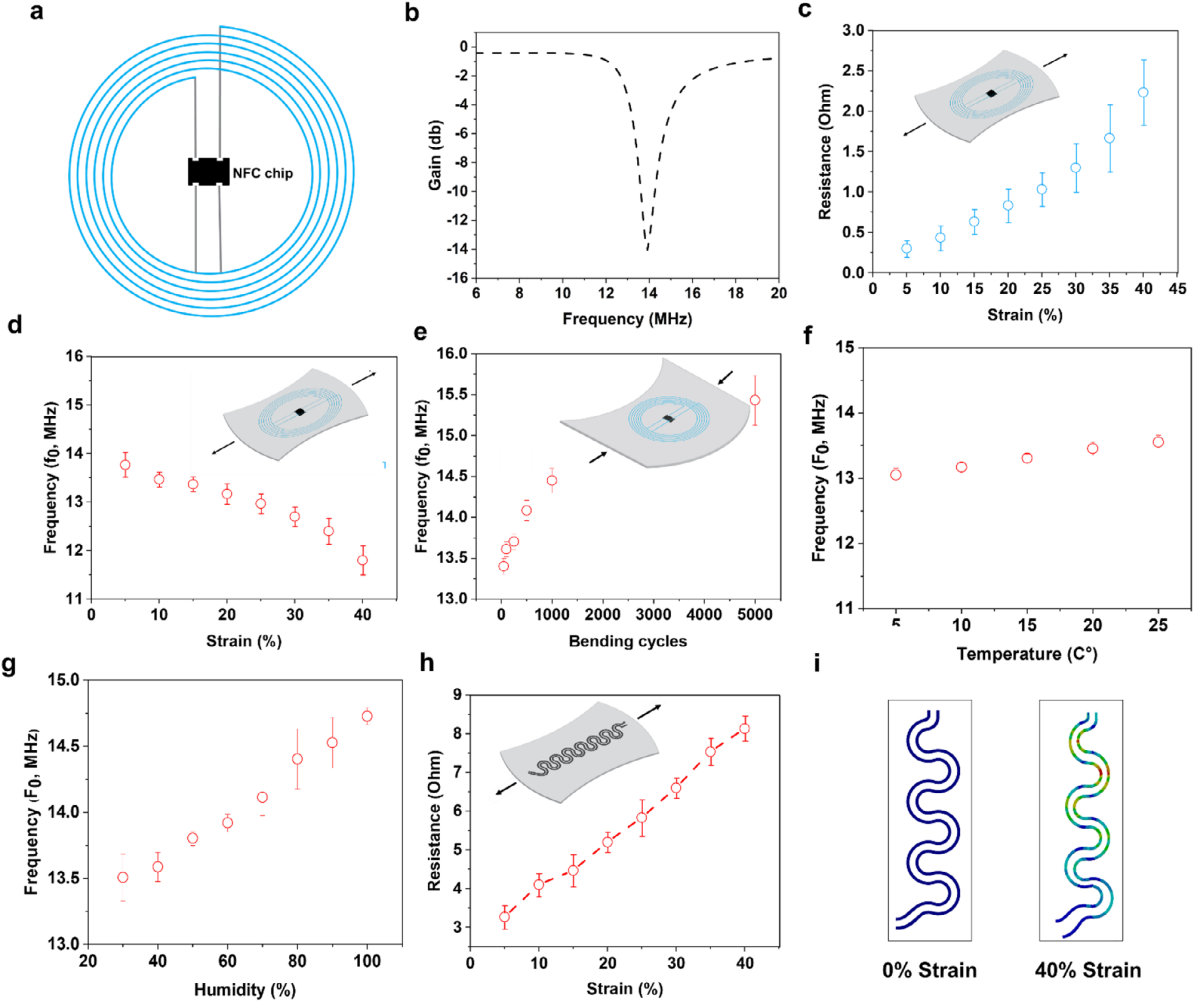
a) An illustration of the NFC antenna with a chip. b) The resonance frequency and the gain of the fabricated antenna. c) The change in the resistance of the antenna coil under a mechanical strain in terms of stretching. d) The effect of mechanical stress in terms of stretching on the resonance frequency of the antenna. e) Bending effect on the resonance frequency of the antenna. f) The effect of temperature change on the resonance frequency of the antenna. g) The effect of humidity change on the resonance frequency of the antenna. h) Strain effect on the resistance of the electrodes, i) ANSYS mechanical simulation of the antenna under different mechanical stress.  Data are expressed as means ± SE.

The third part of the smart packaging is the NFC antenna which is the skeleton of smart packaging. The NFC antenna was employed mainly for two reasons, first to connect both the gas sensor (intelligent packaging) and the releasing mat (active packaging) and hence, to trigger the release of CEO once the food starts to get spoiled. Second, to wirelessly harvest energy and power up the heater integrated inside the mat. **Figure** **3**a illustrates the schematic layout of the NFC antenna along with the chip. Following the fabrication process, the NFC was characterized, as depicted in Figure 3b. The antenna exhibited its peak resonance at ≈14 MHz, while the bandwidth (3 MHz) remained within the standard NFC 13.56 MHz frequency range.

Given that food products are subjected to significant mechanical stress during transportation and shelf storage, it is imperative to investigate the mechanical stability and robustness of the antenna, especially considering its susceptibility to such stresses. Subsequent characterization was conducted after subjecting the antenna and the electrodes to various conditions, including different strain levels (ranging from 0% to 40%), various bending cycles (ranging from 0 to 5000), fluctuations in temperature (ranging from 5 to 25 °C), and changes in relative humidity (ranging from 20% RH to 80% RH), as illustrated in Figure 3c–i. The overall resistance of the antenna conductive traces changed from 0.3 to 2.2 ohms, and the peak resonance frequency altered from 14 to 12 MHz, due to strain variance as depicted in Figure 3c,d, and Figure S5 (Supporting Information). Likewise, under the stress of 5000 bending cycles, the resonance frequency transitioned from 14 to 15.5 MHz, as depicted in Figure 3e. Figure 3f,g illustrates the effects of temperature (changing from 14 MHz to 14.9 MHz) and humidity (shifting from 14 to 15.5 MHz) on the antenna's resonance frequency. It is important to emphasize that although variations in the antenna resonance frequency were observed under different conditions, the antenna's bandwidth consistently remained within the standard NFC 13.56 MHz range (due to 3 MHz), therefore, the overall performance is not significantly affected. On the other hand, the electrodes used to transport the harvested voltage from the NFC antenna showed a. slight increase in the resistance from 3 to 8 Ohm as shown in Figure 3h,i.

### 2.4 Characterization of the Smart Packaging

**Figure 4:**
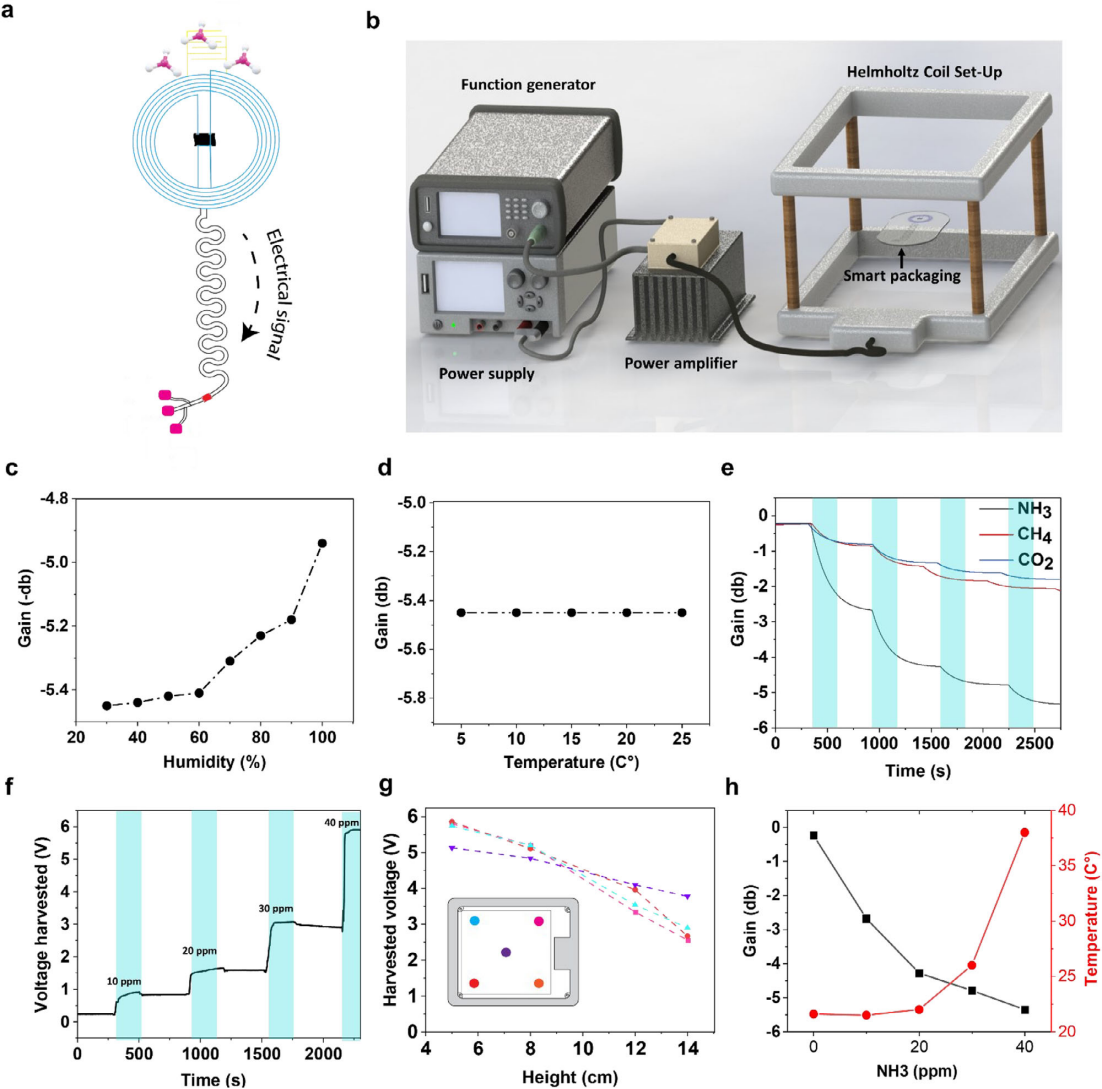
a) An illustration of the final smart packaging composed of a gas sensor, NFC antenna, and active packaging. b) The experimental setup was used to investigate the effect of different gases on the gain and harvested voltage from the NFC antenna. c) The change in the gain of the antenna versus humidity ranging from 30% to 100% to simulate the food packaging environment. d) The change in the gain of the antenna versus temperature ranging from 5 to 25 °C to simulate the storage of the food packaging inside a fridge and at room temperature. e) The gain change of the NFC antenna in the presence of NH_3_, CH_4_, and CO_2_ ranging from 5 to 40 ppm “ammonia”. f) The change in the harvested voltage in the presence of different NH_3_ concentrations ranging from 5 ppm to 40 ppm. g) The change in harvested voltage versus the position inside the Helmholtz Coil. h) The change in Gain and the temperature (active packaging film) versus different concentrations of NH_3_ ranging from 10 to 40 ppm.

**Figure** **4**a shows an illustration of the final device “smart packaging” after the integration of intelligent and active packaging via the NFC. Figure 4b and Figure S6 (Supporting Information) shows the setup used to determine the effects of ammonia on the gain of the NFC antenna. As the smart packaging will be implemented inside food packaging where the temperature and the humidity vary, it was important to investigate the effect of both variables on the gain of the NFC antenna.

Figure 4c,d   illustrates the impact of varying humidity and temperature conditions on the antenna gain in both the OFF and ON states of the smart packaging. Remarkably, the device demonstrates robust stability under humid conditions, attributed primarily to the protective layer of PDMS. This layer acts as an effective moisture barrier due to its inherent hydrophobic properties. Furthermore, the device exhibited excellent stability across a spectrum of temperatures, ranging from 4 °C, representative of refrigeration conditions, to 25 °C, indicative of room temperature environments.

**Figure 5:**
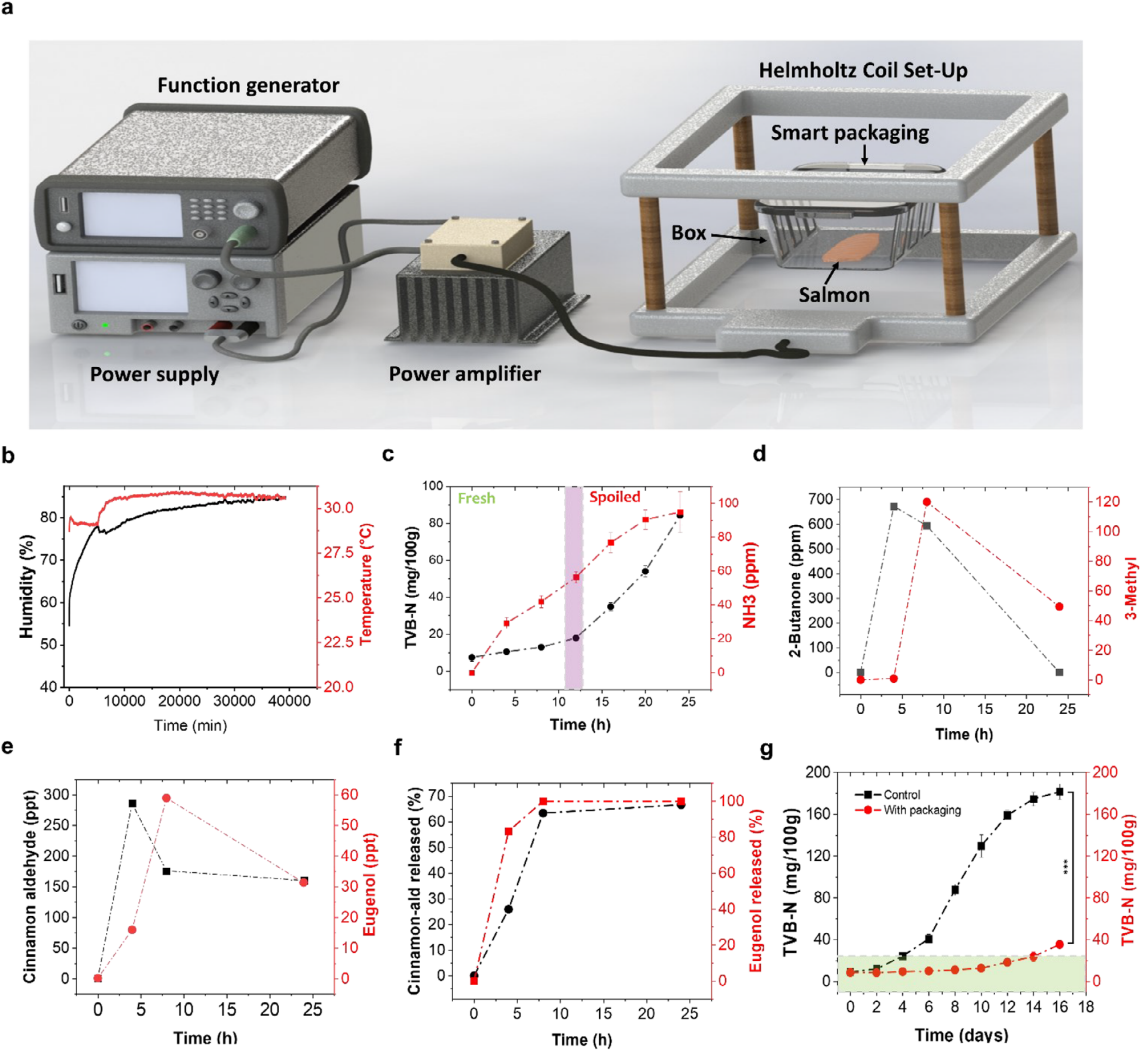
a) The experimental setup used to investigate the performance of the smart packaging with a real sample ‐salmon‐, b) temperature and humidity change inside a box with salmon starting from closing the lid at t = 0 min, c) TVB‐N and NH3 increase inside the box over time, d) 2‐Butanone and 3‐methyl butanol change over time inside a box containing salmon and a powered‐smart packaging measured via GC‐MS, e) cinnamaldehyde, and eugenol change‐over‐time inside a box containing salmon and a powered‐smart packaging measured via GC‐MS, f) cumulative release of cinnamaldehyde, and eugenol over a period of 24 h inside a box containing salmon and a powered‐smart packaging measured via GC‐MS, g) TVB‐N increase at 4 °C with non‐powered “control” and powered smart packaging. Data are expressed as means ± SE; student t‐test for the 16 days point, ***p ≤ 0.001, ns‐not significant.

To simulate the spoilage of fish products and to study the effect of the latter on power harvesting of the NFC antenna in a controlled environment, a gas chamber was used to inject different concentrations of ammonia ranging from 5 to 90 ppm. Figure 4e shows how different gases such as NH_3_, CH_4_, and CO_2_ affected the gain of the NFC antenna. According to the results observed, ammonia had a larger impact on gain, and when concentrations of NH_3_ were increased, gain also increased from 0.4 db to −5.6 db under 90 ppm of NH_3_. Furthermore, the effect of different gases on the gain of the NFC antenna was also examined. As can be seen from the results, an increase in the concentration of CH_4_ and CO_2_ also increased the gain, however, this increase was negligible compared to the one caused by NH_3_, thus illustrating the selectivity of the gas sensor. Figure 4f depicts the variation in wirelessly harvested voltage in response to NH_3_. As the latter concentration augmented from 5 to 40 ppm, the interaction between NH_3_ molecules and the SCNT gas sensor led to an increase in the sensor's resistance. This increased resistance, when incorporated in parallel to the NFC coil, altered the antenna's resonance frequency, bringing it closer to the 13.6 MHz frequency at which the harvested voltage peaks—Consequently, an increase in wirelessly harvested voltage was observed, reaching 5 .8V. This voltage level was sufficient to power up the integrated heater in the active packaging, thereby initiating the release mechanism. As a point of clarification, the concentration of NH_3_ in this experiment was chosen to simulate the spoilage of a food product, in other words, an increase in the concentration of NH_3_, which represents the spoilage of the fish, leads to an increase in the voltage harvested, which is then used as a switch to trigger the release of the antioxidant.

Another factor affecting the harvested voltage was the position of the smart packaging inside the Helmholtz Coil Set‐Up on the three axes, so it was important to optimize the position of the device inside the set‐up as shown in Figure 4b. The results showed that the inner side of the edges of the box and a distance of 5 cm of the Helmholtz Coil gave the highest output (Figure 4g).

Figure 4h illustrates the relationship between gain and temperature increase in response to varying NH_3_ concentrations. In the absence of NH_3_, both the gain and the temperature of the active packaging remained at 0.2 dB and 20 °C, respectively. However, as the NH_3_ concentration gradually increased, the gain of the NFC antenna increased, while the temperature remained unchanged. Furthermore, once the NH_3_ concentration reached 30 ppm the gain dropped to ≈−5 dB, corresponding to a harvested voltage of 3 V, and the temperature of the active packaging film was increased to 27 °C by harnessing the ohmic heating. Subsequently, when the NH_3_ concentration reached 40 ppm, the harvested voltage increased to 5.8 V, and the temperature of the active packaging film rose to 37 °C, a critical threshold for triggering the release mechanism.

### 2.5 Validation of the Smart Packaging

The practical application of smart packaging was tested with fish in real conditions. **Figure** **5**a illustrates the experimental setup, salmon and smart packaging were placed at the bottom and top of a box, respectively. Then, a headspace sample was extracted using an SPME fiber and analyzed by GC/MS. Total Volatile Basic Nitrogen (TVB‐N) plays an important role in assessing the quality of meat products and is considered a gold standard. When TVB‐N reaches 25 mg per 100 g of salmon, deterioration is identified.^[^ ^41^ ^]^ Hence, to correlate at which NH_3_ concentration the salmon begins to deteriorate, TVB‐N values were calculated while measuring NH_3_ concentration at room temperature using a commercial metal oxide gas sensor (Figure S9, Supporting Information). At RT, the initial concentration of TVB‐N was 1.3 ± 0.4 mg, which increased after 9 h of storage to 20.3 ± 0.01 mg. After 16 h of storage, the amount of TVB‐N increased above the permitted limit across all samples with a corresponding NH_3_ concentration of 60 ppm. In light of this result, an NH3 threshold of 40 ppm was selected to trigger the release of the CEO (Figure 5c).

The smart packaging system relies on CA and EG releases to extend salmon shelf life. Monitoring the concentrations of these antioxidants and spoilage markers like 2‐butanone and 3‐methyl butanol was essential (Note S2, GC/MS In Supporting Information). Figure 5d,e shows changes in spoilage markers and antioxidants in the headspace. Initially, at t═0, all markers were absent, indicating the fish's freshness. However, after 4 h, concentrations of 2‐butanone, cinnamaldehyde, and eugenol increased to 660, 270, and 70 ppm, respectively. The rise in 2‐butanone signaled the beginning of spoilage, which in turn triggered the release of antioxidants, as evidenced by the presence of cinnamaldehyde and eugenol. After 24 h, the concentrations of the markers decreased to 0, 45, 160 ppm, and 32 ppm for 2‐butanone, 3‐methyl butanol, cinnamaldehyde, and eugenol, respectively. These results demonstrate that fish spoilage was effectively prevented, as evidenced by the reduction in spoilage marker concentrations. However, the persistently high levels of antioxidants in the headspace suggest that not all cinnamaldehyde and eugenol were depleted, indicating the potential for longer fish preservation. Furthermore, Figure 5f reveals the cumulative release after 24 h of CA and EG, with 67% and 100%, respectively, likely due to its lower concentration compared to cinnamaldehyde.

The smart packaging's performance was subsequently studied at 4 °C. The assessment at the lower temperature focused on measuring TVB‐N levels to monitor fish freshness, instead of relying on VOCs. After four days of storage, the control sample (non‐powered smart packaging) exhibited a significant increase in TVB‐N levels to 32 ± 0.8 mg, surpassing the acceptable threshold of 25 mg per 100 g of sample. In contrast, even after 14 days, the samples with smart packaging consistently maintained TVB‐N levels below the permissible limit, indicating effective preservation (Figure 5g). Table S3 (Supporting Information) depicts a radar chart with a comprehensive evaluation of the smart packaging system developed in this work by comparing its performance across multiple criteria against existing packaging reported in the literature. The comparison suggests that the smart packaging in this work performs robustly in several key areas. However, improving biodegradability and shelf‐life extension is still needed.

In conclusion, our study presents a breakthrough in food packaging with the development of a battery‐less, autonomous smart packaging system. This system effectively combines real‐time food monitoring and the controlled release of active compounds to significantly extend the shelf life of food products. Employing a gas sensor for freshness monitoring and wirelessly powering the system via an NFC antenna allowed for a controlled release of active compounds, moreover, the system demonstrated the capability to dynamically respond to food spoilage, thus addressing both performance and sustainability challenges in food packaging. The successful extension of food shelf life up to 14 days in our tests underlines the potential of this system as an innovative IoT solution in the food industry, making a substantial contribution to global efforts to reduce food waste and improve sustainability. This research opens new avenues for smart packaging solutions that can adapt and respond to changing food environments, paving the way for a new era of efficiency in food storage and distribution. However, future studies should include the investigation of the exact release mechanism of active compounds as well as the effect of CEO on the organoleptic properties of the food product.

## Conflict of Interest

The authors declare no conflict of interest.

## Author Contributions

A.D. performed conceptualization, visualization, and methodology, and wrote, reviewed, and edited the original draft. A.D., A.M., E.L., G.W., R.R., S.I., T.N.T., and E.L.P. performed investigations and wrote, reviewed, and edited the original draft. P.L., L.P., E.B., and A.A. performed the supervision and wrote, reviewed, and edited the original draft.

## Supporting information



Supporting Information

## Data Availability

The data that support the findings of this study are available from the corresponding author upon reasonable request.
