# Supplementary material for: Battery‐Free, Stretchable, and Autonomous Smart Packaging
Source: Adv Sci (Weinh). 2025 May 12;12(22):2417539. doi: 10.1002/advs.202417539 (PMC12165031; doi:10.1002/advs.202417539)
Supplement: Supplementary file 1 — Supporting Information [file ADVS-12-2417539-s001.docx]

**SUPPORTING INFORMATION**

**Battery-free, stretchable, and autonomous smart packaging**

Ali Douaki ^a d e*^, Mukhtar Ahmed ^a f^, Edoardo Longo ^b^, Giulia Windisch ^b^, Raheel Riaz ^a^, Sarwar Inam ^a^, Thi Nga Tran ^c^, Evie L. Papadopoulou ^c g^, Athanassia Athanassiou ^c^, Emanuele Boselli ^b^, Luisa Petti ^a *^, Paolo Lugli ^a *^

*^a^  Sensing Technologies Laboratory (STL), Faculty of Engineering, Free University of Bozen-Bolzano, Piazza Università 5, 39100, Bozen, Italy.*

*^b^ Faculty of Agricultural, Environmental and Food Sciences, Free University of Bozen-Bolzano, Piazza Università 5, 39100, Bozen, Italy.*

*^c^ Smart Materials Group, Istituto italiano di Tecnologia, via Morego 30, 16163 Genova, Italy*

*^d^ Optoelectronics Research Line, Istituto italiano di Tecnologia, via Morego 30, 16163 Genova, Italy*

*^e^ Dip. di Scienze e Metodi dell'Ingegneria, Università di Modena e Reggio Emilia, via Amendola 2, 42122 Reggio Emilia, Italy.*

*^f^ ABB Corporate Technology Center, Krakow, Poland.*

*^g^ Bedimensional SPA, Lungotorrente Secca, 30R, 16163 Genova, Italy.*

**Materials and Methods**

***Materials***
Ink pastes, silver chloride ECI 6038E, and silver ECI 1011, were purchased from LOCTITE E&C (CA, USA). Polypropylene carbonate (PPC) was purchased from Empower (USA). Cinnamon essential oil of the Cinnamomum Verum species was purchased from Maitreya-Natura (Italy). Acetone, ethanol, PNIPAM, N, N′-methylene bisacrylamide (MBA), Irgacure 2959 (was used as the photoinitiator (PI)), PEDOT:PSS were purchased from Sigma-Aldrich. All chemicals used in this work are analytical grade and were used without any further purifications.

***NFC antenna and circuits***

The spiral antenna (50 mm diameter, 8 turns, and width of 0.8) was designed and simulated in Ansys HFSS. The simulations (Ansys HFSS 20 User’s Guide, Ansys Inc., 2020) used a lumped port to define the S11 and the port impendence (Z). The simulation yielded the scattering parameters (S11), and the inductance (L). The simulated antenna was fabricated by first fabricating polylactic acid (PLA) 3d printed positive mold was used to fabricate the device, PDMS (10:1) was cast and degassed then cured overnight at room temperature and pealed-off. Afterward, a stretchable silver ink was used to fill the cavities, then the NFC chip (M24LR16E) was connected and the whole device was cured at 120 C°. The connections between the top and bottom layers were manually established with copper wire and silver glue.

***Fabrication of the CNT gas sensor***

To prepare the SWCNTs gas sensor, first, the IDE with a spacing of 300 µm were fabricated by screen-printing (with an automatic screen-printer AurelC920, Italy) a silver (Ag) ink paste (ECI 1011 from LOCTITE) on a poly(diméthylsiloxane) (PDMS) substrate (with already fabricated NFC antenna and electrodes), followed by a curing step at 120 °C for 15 min. Afterward, a water-based SWCNTs dispersion was then spray coated using an automated system equipped with an industrial air atomizing spray valve (Nordson EFD, USA) on top of the printed electrode. Finally, a PDMS layer with a thickness of ≈120 um was spin-coated, to protect the sensing film from humidity.

***Preparation of controlled release mat***

Polypropylene carbonate was dissolved in acetone using varying solvent ratios (1:9, 2:8, and 3:7 v/v) at a concentration of 10 wt%, and then stirred for 2 hours. To encapsulate cinnamon essential oil (CEO) within the PPC fibers, 5% v/v CEO was mixed into the PPC solution (PC5). The mixtures were thoroughly vortexed before electrospinning to ensure uniformity. The electrospinning was conducted in an ambient environment. PC5 solution was transferred into a plastic syringe, which was then placed in a syringe pump (NE-1000 New Era Pump Systems, Inc.), delivering a flow rate between 250 and 650 mL/h. An 18 kV voltage (supplied by EH40R2.5, Glassman High Voltage, Inc.) was applied between the 18-gauge stainless-steel spinneret and an aluminum collector situated 25 cm away. Following electrospinning, the PC5 mat was left on the aluminum foil and immersed in a 3% w/v PEDOT: PSS solution for 30 seconds, then air-dried. PNIPAM hydrogels were initially prepared by blending 6% (w/v) NIPAM, 0.3% (w/v) N,N-methylene-bis-acrylamide (MBA), and 0.5% (w/v) of PI solution in water. The mixture was degassed with N2 for 30 minutes and then polymerized under UV light for 4 minutes. The UV lamp was positioned 10 cm above the solution, emitting an intensity of 650 mW. After polymerization, the hydrogels were dissolved in acetone and subjected to tip-sonication for 2 minutes to produce PNIPAM particles. Finally, the PC5P mat was gently removed from the aluminum foil, and the PNIPAM solution was spin-coated onto the lower layer of the PC5P mat at 1000 rpm for 30 seconds.

***SWCNT gas sensor characterization***

The sensor characterization was performed in a custom-made gas chamber by exposure to continuous constant gas flux (500 mL/min) at different gas targets (NH_3_, CH_4_, and CO_2_) concentrations. The desired concentrations were achieved by dilution of the target gas ammonia with a carrier gas (air). A measurement cycle was composed of a sensing interval, where the device was exposed to the desired gas target concentration for 300s. Afterwards, a recovery interval (to desorb the trapped gas molecules on the SWCNT film was performed by heating the sensor to 60 °C for 900 s by using a Peltier element. Finally, increase the cooling rate of the sensor and for the extraction of residual gas molecules, a high flux of air (1000 mL/min) was kept for another 300 s at room temperature, then another 300 s cycle of air with the same flux used during the next sensing interval. All the measurements were automated with the use of LabVIEW 2019 which controlled the Flow Meter Units, Source meter 2602b (Keithley, USA), and Digital Multimeter 6510 (Keithley, USA).

***Controlled release mat characterization***

Scanning Electron Microscopy (JEOL JSM-6490LA) was used to characterize the morphology of the electrospun fibers. For the analysis of the fabricated heater, a thermal camera and a temperature sensor (Pt-100) were used for in-situ temperature monitoring. The heaters were driven by a source meter unit (Keithley 2602b Dual-channel SMU) and the resistance was measured with a Digital Multimeter (Keithley 6510). All measurements were automated with LabView 2019.

**Mechanical tests**

The mechanical properties of the fiber mats were analyzed by performing stress–strain curves using a dual column tabletop universal testing System Instron 3365, with 50 mm/min−1 cross-head speed. Samples were cut in dog-bone shape with an effective length of 25 mm. An average of 3 measurements were taken for each sample.

***Electromagnetic characterization***

Electromagnetic properties of the antenna were measured with an impedance analyzer (Keysight E4990A, USA) over a frequency range of 1–20 MHz. The resonant frequency of the NFC antenna was measured with a network analyzer (Keysight E5063A) by placing the device at the center of the primary coil at a vertical distance of 3 cm.

***Mechanical stress simulation***

Ansys workbench 2021 software was used to simulate the mechanical stress on the antenna and the serpentine wire. The composite layer (PDMS and Ag) consisted of a 6-node linear triangular prism (C3D6). The simulations implemented values of the elastic modulus of PDMS and stretchable silver ink.

***Mechanical Characterization***

The antenna, CNTs gas sensor, and serpentine performance under mechanical stress were characterized under strain and bending deformation using a custom-made cyclic bending setup. Using two parallel clamps (one fixed and one movable) and controlled with a LABVIEW program 2017 (NI, TX, USA). The samples were mounted in the clamps, Afterward, it was flattened and bent down to 6 mm of bending radius for multiple cycles. The performance of the antenna and serpentine was evaluated in terms of resonance frequency and resistance after a certain number of cycles (50, 100, 250, 500, 1000, and 5000 cycles).

***Transmitted Coils and Electronics***

The transmitted coil (ID ISC.ANT310/310) was bought from FEIG Electronic, used at 13.56 MHz with the help of a Network Analyzer (HP 8753E). The power source consisted of a signal generator (Keysigh 33522A), a power amplifier (Mini-Circuits ZHL-100W-52X-S +), a laboratory power supply (Elektro-Automatik EA-PS 3032-10B).

Migration test

The overall migration (OM) of molecules from the samples was performed using ethanol:water (90:10) as fatty food stimulant, along the EU Technical Guidelines for compliance testing in the framework of the plastic FCM Regulation (EU) No. 10/2011.

The samples were immersed in a vial containing 20 mL of ethanol:water solution (90:10 v/v) and placed in an oven at 70 °C for 2 h. Then the samples were taken out and the overall migration into the fatty food simulant was calculated using the following equation,

$$M=\frac{(m0-mf)(1000)}{S}$$

where, in this case, m_0_ and m_f_ are the initial and final mass of the sample.

**Climate chamber**

For investigating the effect of humidity and temperature of the gain of the antenna, Espec SH-262 Environmental Chamber was coupled to a network analyzer (Keysight E5063A) and were both controlled using LabVIEW program.

***Measurement of Power Distribution in the Helmholtz Coil Set-Up***

The electrical field distribution and thus the special influence on the wirelessly transmitted electrical power was measured in the active chamber in a single coil configuration. To measure the harvested power a Source meter 2602b (Keithley, USA) was used. During the measurements, the NFC antenna was placed parallel to the transmitter coils.

**TVB-N measurement and NH_3_**

The Total Volatile Basic Nitrogen (TVB-N) content in the fish sample was assessed using a semi-micro nitrogen determination method, as detailed by Wang et al. (2018). Each fish sample served as a unique biological replicate, leading to greater variability in the data compared to a scenario where a single fillet is homogenized and divided into multiple samples for replication.


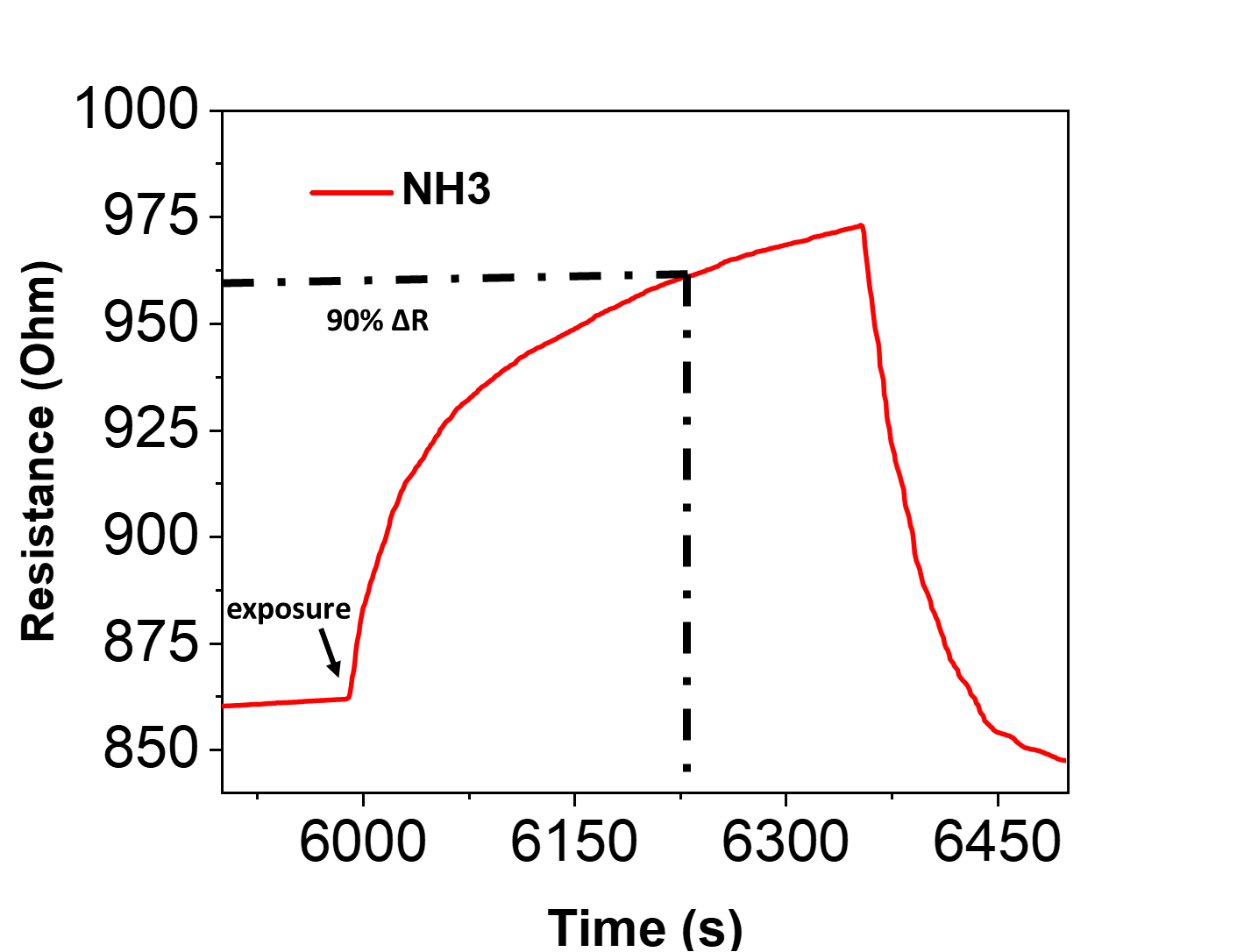


**FIG. s1|** Response time of the SWCNT gas sensor.


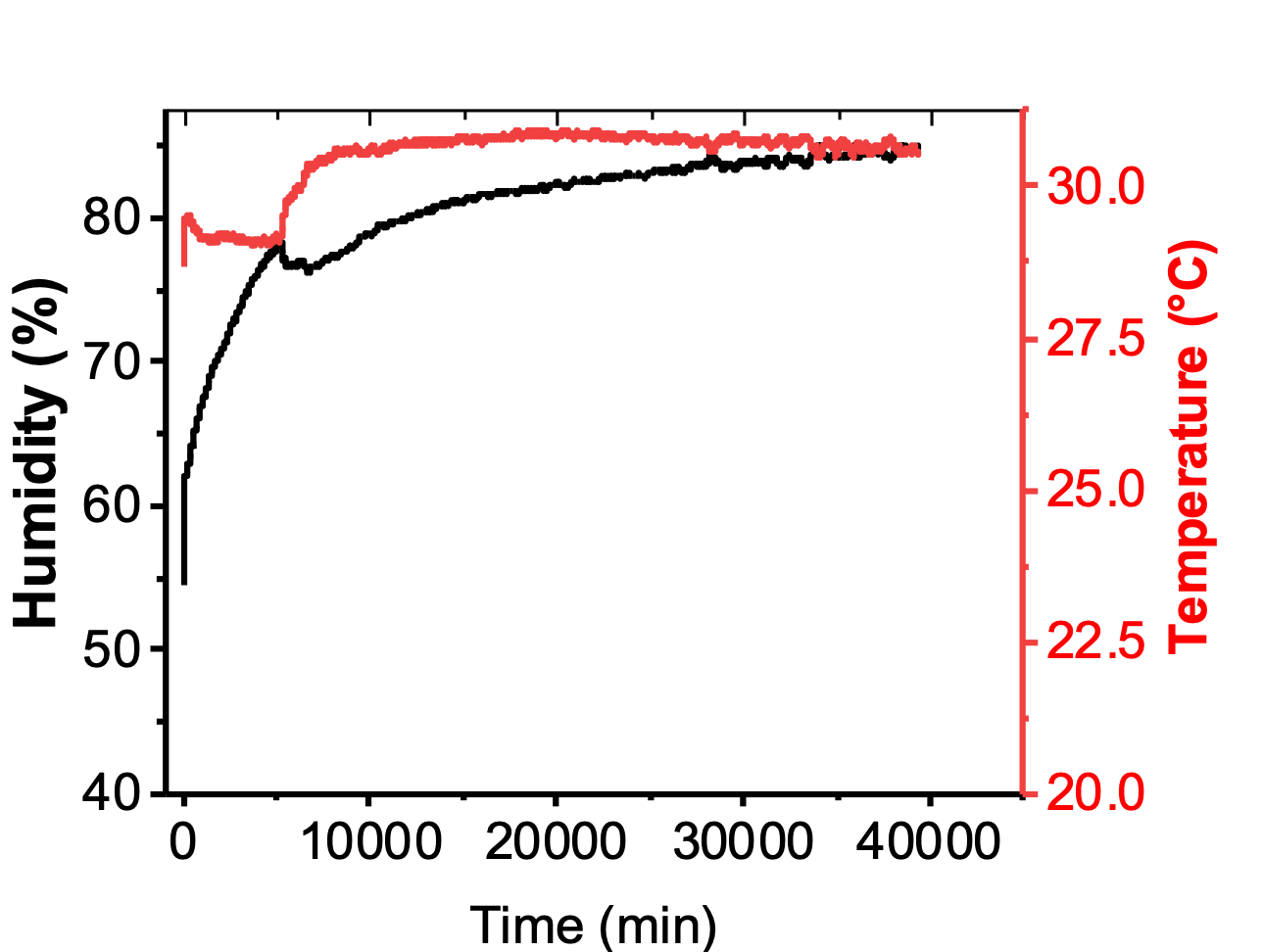


**FIG. s2|** Temperature and humidity change inside a box with salmon starting from closing the lid at t=0 min.

**Note 1.** Release device in the smart food packaging

The second component of the smart packaging system that we describe here is the part that releases the active compound upon food spoilage. Essential oils, particularly CEO, have been extensively studied for their antimicrobial and antioxidant properties, making them promising candidates for food preservation and pharmaceutical applications^[1]^. Among the various bioactive compounds present in CEO, cinnamaldehyde (CA) and eugenol (EG) are the most dominant, as consistently reported in the literature and confirmed by gas chromatography–mass spectrometry (GC-MS) analysis (10.3390/foods12020332) ^[2,3]^.

For this purpose, a flexible, thermo-responsive device engineered for controlled and on-demand release of bioactive compound was developed. The interested reader can find the full characterization of this component in our previous publication ^[4]^. In short, the device is composed of polypropylene carbonate (PPC) electrospun, fibrous mats, with cinnamon essential oil (CEO) encapsulated in the fibers, acting as the bioactive compound. The bottom side of the fibrous mat is covered with an electrically conductive PEDOT:PSS layer, serving as an electrically conductive layer. The top side is covered with a thermo-responsive polymer PNIPAM layer, that controls the release of the CEO (as shown in Fig. s4) ^[4]^. The SEM micrographs of PPC fibers, cross-section of PNIPAM film, and cross-section of the final device are shown in Fig. s4b, s4c and s4d, respectively, highlighting the compact structure of the three different layers upon the addition of PEDOT:PSS and PNIPAM.


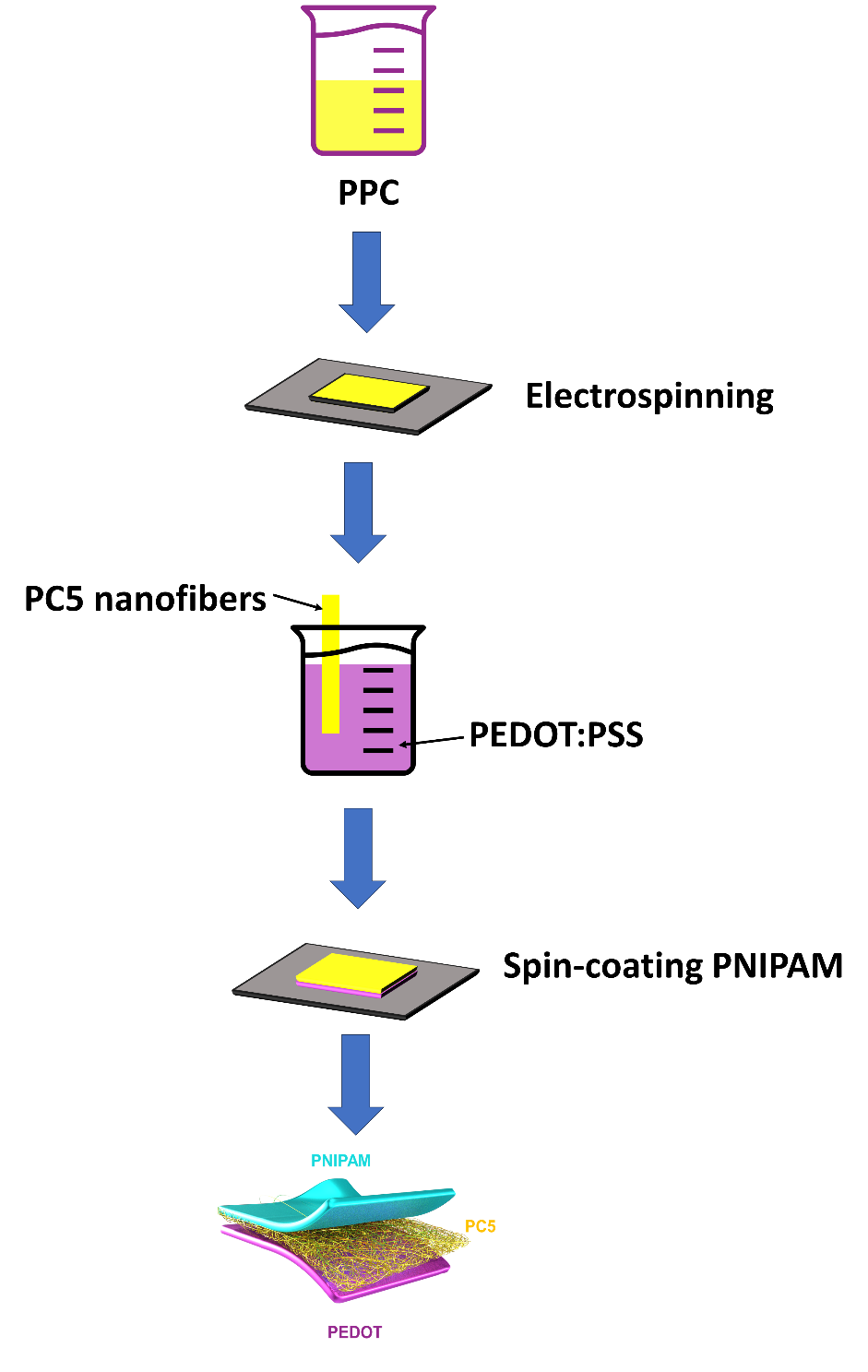


**FIG. s3|** Fabrication process of the active packaging.

To confirm the release and the profile of encapsulated CEO once the temperature of the device is raised above the LCST value, the latter was investigated using gas chromatography/ mass spectrometry GC/MS. To start the release a potential of 3 V was applied across the mat and the device was kept ON for two hours to trigger the release and then OFF for two hours. Every two hours a sample was extracted from the headspace of the box and analyzed with GC/MS. As the cinnamaldehyde (CA) makes up to 80% of the composition of the CEO, it was selected as a marker of the release and was monitored (Fig. s4f) ^[1]^. The release profile showed a steady release of around 10% ± 4 every time the voltage was applied over the range of 28 h. After 28 h, the PC5PP sample released up to 58.78 ± 1.25% of their total initial amount of CA and importantly, there was almost no leakage when the device’s temperature was kept below the LCST, hence, demonstrating the potential of the device for precise controlled release of bio-compounds and therefore the feasibility to integrate it into the smart packaging) ^[1]^. The release profile showed a steady release of around 10% ± 4 every time the voltage was applied over the range of 28 h. After 28 h, the PC5PP sample released up to 58.78 ± 1.25% of their total initial amount of CA and importantly, there was almost no leakage when the device’s temperature was kept below the LCST, hence, demonstrating the potential of the device for precise controlled release of bio-compounds and therefore the feasibility to integrate it into the smart packaging.


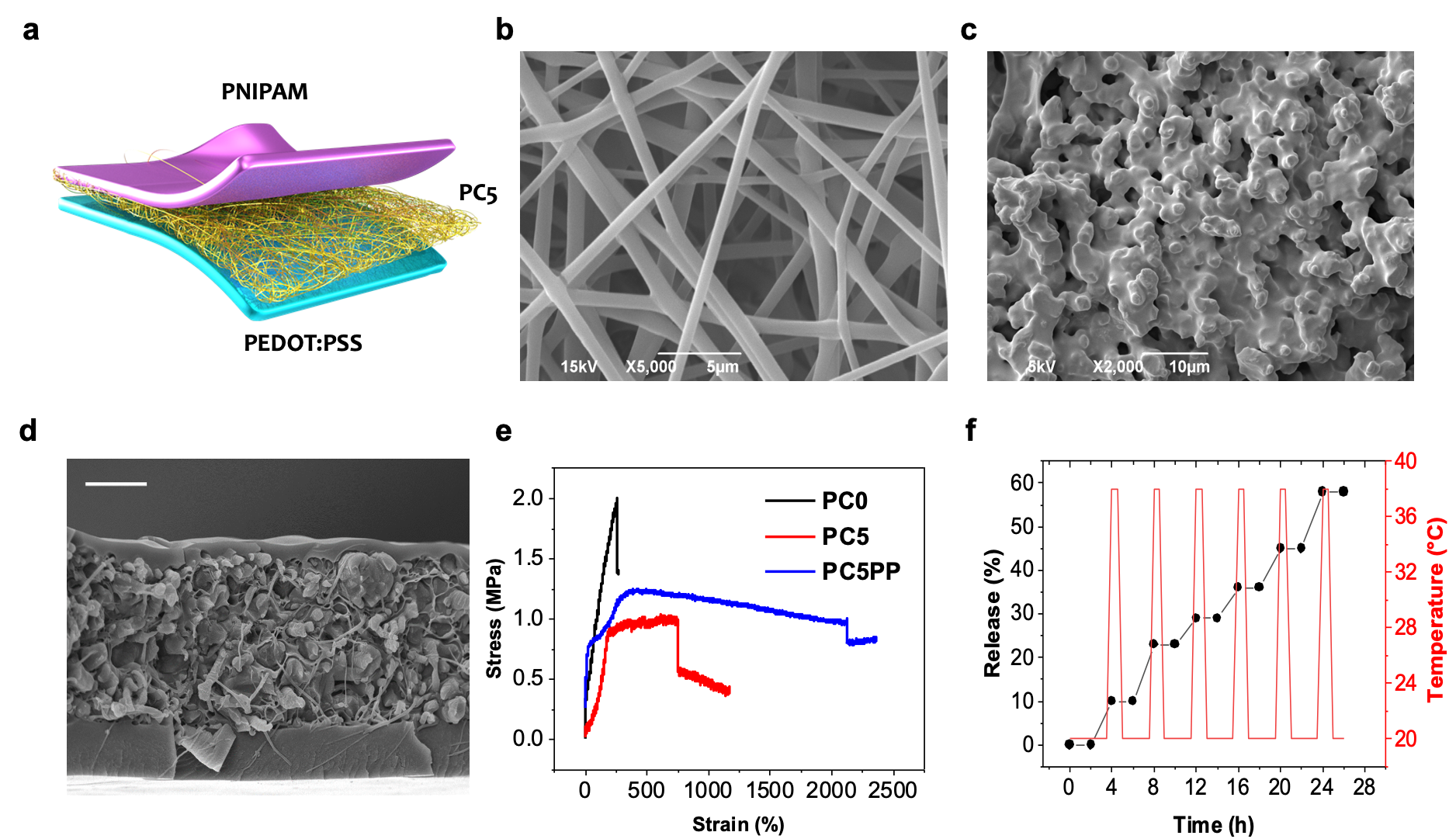


**Fig. s4|** (a) An illustration of the three layers forming the active packaging, (b) SEM image of PPC nanofibers used as a substrate and for encapsulating the active compounds, (c) SEM image of the PNIPAM film, (d) SEM cross-section of the active packaging, scale 10 um, (d) Stress vs strain of the different layers of the active packaging. (f) The release profile of the cinnamaldehyde upon an applied voltage of 3V over a period of 24 h.

**Table. S1|** Nomenclature of different active packaging layers.

| Compound Name | Acronym |
| --- | --- |
| polypropylene carbonate | PC0 |
| polypropylene carbonate/5% CEO | PC5 |
| polypropylene carbonate/5% CEO/PEDOT:PSS | PC5PP |
|  |  |
|  |  |
|  |  |
|  |  |
|  |  |


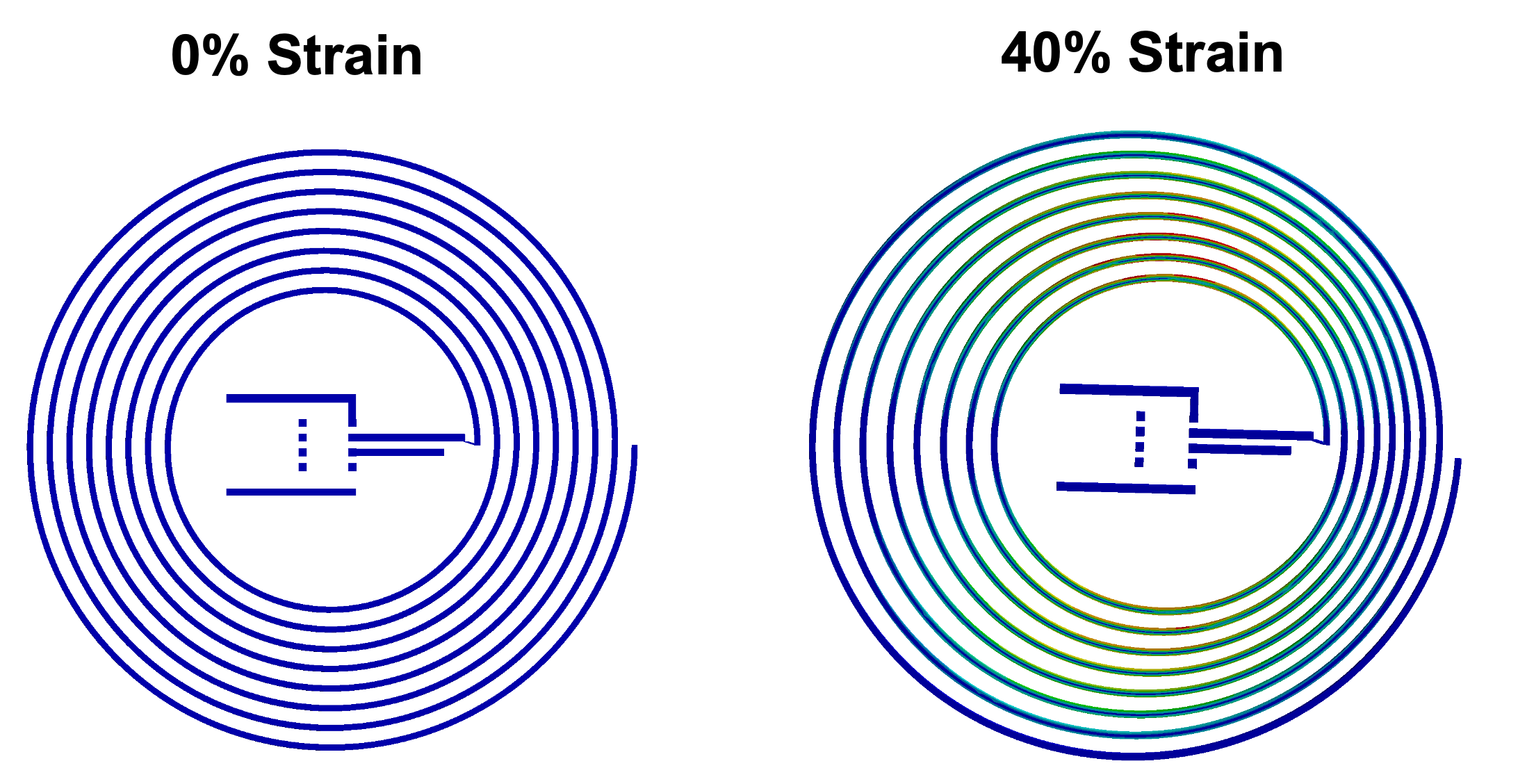


**FIG. s5|** ANSYS mechanical simulation of the antenna under mechanical stress.


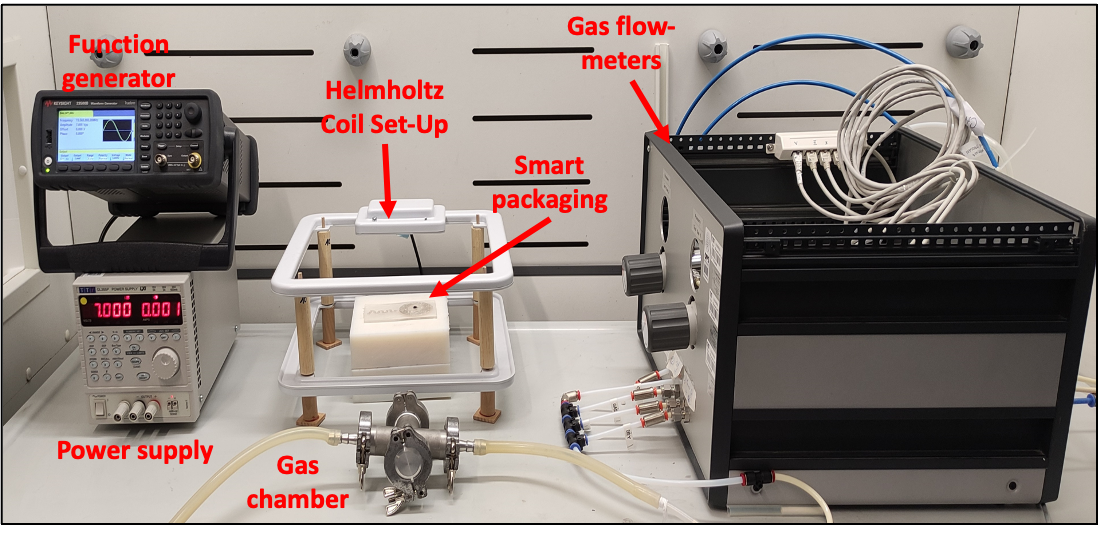


**FIG. s6|** Optical image of the setup used to characterize the smart packaging in a gas chamber.

Fig. s7 illustrates the effect of the size of the active packaging on the amount of energy required to raise the active packaging film above the LCST value (32°C). Each of 1 cm^2^, 0.25 cm^2^, and 0.06 cm^2^ of active packaging films required 63 mW, 22 mW, and 2 mW, respectively, to power the heater above the LCST value, hence, an active packaging film of 0.06 cm^2^ was used. Since the controlled release of the CEO relies on the harvested voltage and its ability to raise the temperature above the LCST value, it was essential to study the effect of increasing NH_3_ on the temperature rise of the active packaging film


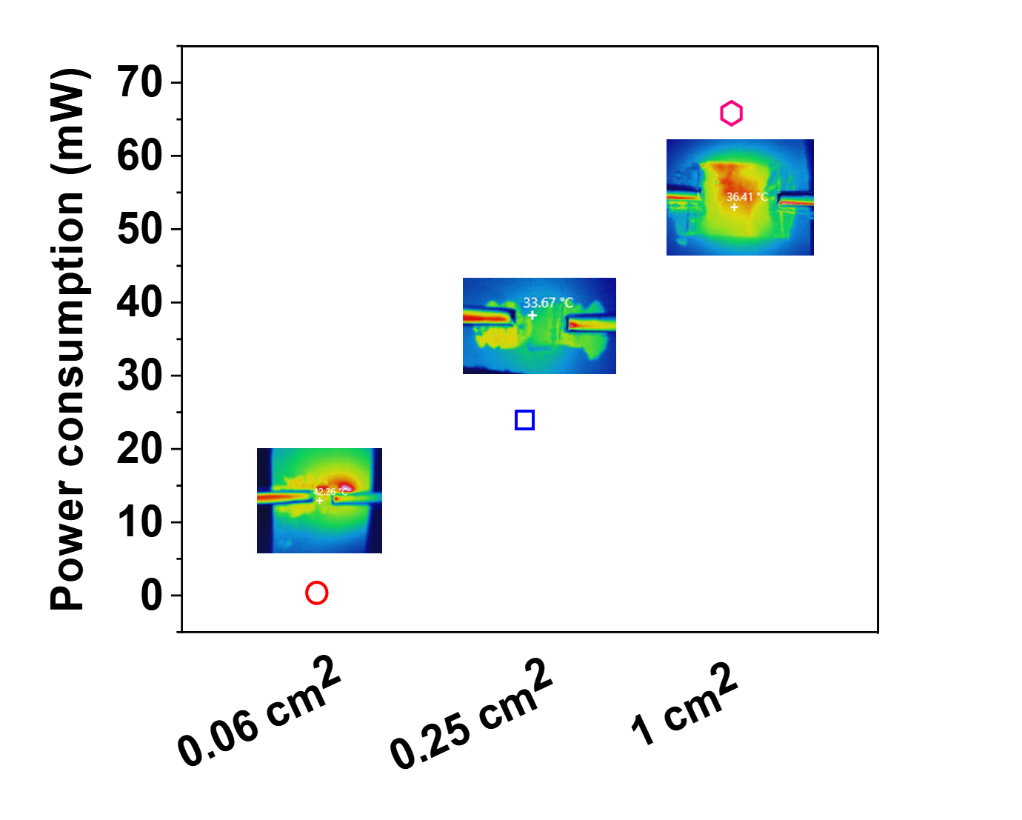


**FIG. s7|** The effect of the active packaging size on the power needed to reach 35 °C.

Since the smart packaging will be in contact with food, it is crucial to study the migration of compounds from food packaging into the food. Therefore, food migration tests were conducted using 20 mL of an ethanol:water solution with a volume ratio of 90:10, simulating a fatty food stimulant ^[4]^. For these tests, samples without cinnamon essential oil were used to ensure that the release of essential oil would not influence the results. As depicted in Fig. s8, the overall migration of the final smart packaging remained below the limit of 10 mg/dm^2^, as defined by the current legislation (Commission Regulation (EU) No. 10/2011), demonstrating the safety of the smart packaging for food contact.

**FIG. s8|** Migration test of the final smart packaging device (gas sensor, NFC antenna, and active packaging).


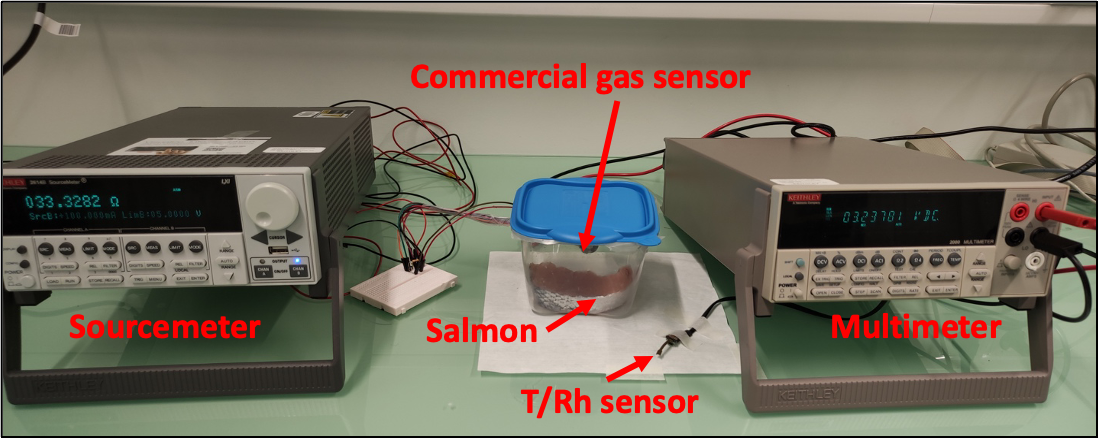


**FIG. s9|** Optical image of the setup used to measure NH3 concertation during salmon spoilage.

**Note 2.** GC/MS

**Sample preparation:**

Fresh salmon was bought at a local supermarket and then transported to the laboratory for analysis on the same day of purchase. Upon arrival, the fish were immediately filleted into 25 g samples. Afterwards, they were placed into individual sterile polyethylene (500 ml) and carefully enclosed boxes. For volatile compound extraction, a solution of 2-octanol (utilized as an internal standard) was introduced into the headspace. Subsequently, a fiber (SPME, 1 cm, 23 Ga, DVB/CAR/PDMS, Supelco) was employed to extract volatile organic compounds from the headspace over the salmon samples and extraction procedures were conducted at room temperature for a duration of 30 min.

The GC/MS method was adapted from a published report ^[5]^. Each analysis was carried out with manual injection on an Agilent 7890A gas chromatograph coupled to an Agilent 5975 quadrupole mass detector (Agilent Technologies Italia SpA, Cernusco sul Naviglio, Milano, Italy). The thermal desorption of the SPME fiber took place in the GC inlet at 240 °C for 6 min. The separation was performed on a MEGA-WAX Spirit column (0.30 µm/0.18 mm/40 m) in split mode (1:10). Helium carrier gas flow rate was 0.7 mg·L^−1^ (constant flow, average velocity 27.022 cm/sec). The temperature program was: 40 °C for 0 min, 40–172 °C at 1.5 °C min−1, then 172–240 °C with 10 °C min−1 rate, and finally 3 min at 240 °C. The mass spectrometer operated in EI+ mode at 70 eV. The mass range was m/z 34–360 at 1 spectrum·s−1; the temperature of the ion source and quadrupole were 230 °C and 150 °C, respectively. The total ion current (TIC) peaks were expressed as relative abundances (peak areas) and were manually aligned. The peaks were assigned by comparison with reference mass spectra (NIST 2011 database).

It is important to highlight that this experiment was conducted exclusively at RT. This decision was taken considering the crucial role of VOCs sampling needed for the GC/MS in the characterization process. Indeed, at 4°C, the volatility of VOCs diminishes significantly, therefore, this reduced volatility hinders the effectiveness of GC/MS, thereby the experiments were performed at RT.

After establishing the appropriate triggering threshold, the next step involved selecting markers for salmon spoilage. For this purpose, GC/MS was employed to investigate the released VOCs during the salmon spoilage. Among the many VOCs detected, two were chosen as representative markers due to their established significance as spoilage indicators in prior studies, and their retention time was not overlapping with those of cinnamaldehyde and eugenol (as shown in Fig. s14) ^[6–8]^. Consequently, 2-butanone and 3-methyl butanol were identified and selected as key VOC markers. As has been previously investigated, the production of 2-butanone and 3-methyl butanol during fish spoilage is a result of different biochemical processes. 2-Butanone is mainly produced from the oxidative degradation of lipids, catalyzed by both enzymatic activities and bacterial metabolism (*Psychrobacter* and *Pseudoalteromonas*). On the other hand, 3-methyl butanol is typically produced from the catabolism of proteins, more specifically, the amino acid leucine, by microbial enzymes such as those from Pseudomonas ^[6–9,9]^. ^[6,9,10]^

**FIG. s10|** GC-MS calibration curve for cinnamon aldehyde.

**FIG. s11|** GC-MS calibration curve for eugenol.

**FIG. s12|** GC-MS calibration curve for 2-butanone.

**FIG. s13|** GC-MS calibration curve for 3-methyl butanol.


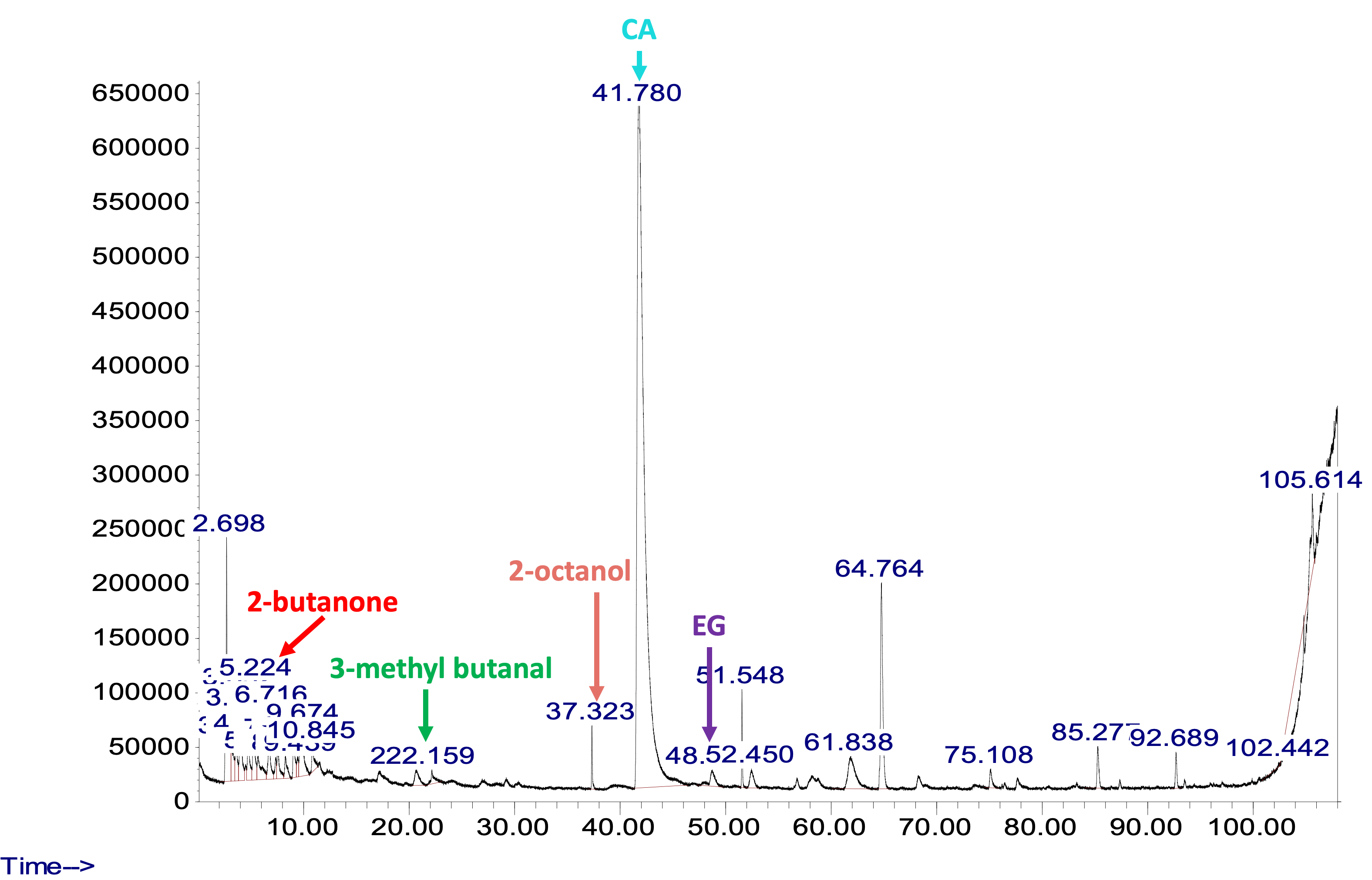


**FIG. s14|** GC-MS chromatogram of spoiled Salmon with CEO, showing the two spoilage markers 2-butanone and 3-methly-1-butanol, CEO markers (cinnamon “CA” aldehyde and eugenol “EG”), and the internal standard (2-octanol)

**Table. s2|** Compounds identified in a spoiled salmon by GC/MS analysis.

| Compound Name | Retention Time (min) |
| --- | --- |
| Methyl thiol | 2.0 |
| 2-Heptanone | 2.9 |
| 2-butanone | 5.2 |
| Dimethyl disulfide | 12.6 |
| 3-methyl-1-butanol | 22.21 |
| Acetone | 29.0 |
| Hexanone acid | 25.3 |
| 2-nonanone | 38.29 |

FIG. s15| 2-Butanone and 3-methyl butanol change over time inside a box containing salmon (control sample) measured via GC-MS.

**Table. S3|** C*omparison between this work and the food packaging reported in literature*

| work | Shelf-life | Biodegradable | Sensing | Scalable | Soft | Self-powered | Stretchable | Closed system |
| --- | --- | --- | --- | --- | --- | --- | --- | --- |
| This work | 12 | No | x | x | x | x | x | X |
| ^[11]^ | 15 | x | No | x | x | No | No | No |
| ^[12]^ | 0 | x | No | x | x | No | X | No |
| ^[13]^ | 0 | No | x | x |  | x | No | No |
| ^[14]^ | 8 | No | x | x | x | x | No | No |

Note 3. EstimateEconomic analysis

The reported system reported in this work was designed for scalability, leveraging cost-effective fabrication methods such as screen printing, spray-coating, and electrospinning for key components (PEDOT:PSS, PNIPAM, and the CNT-based gas sensor). Both the gas sensor and NFC antenna can be produced via roll-to-roll processing, a high-throughput technique that enables low-cost mass production.

Moreover, industrial-scale manufacturing will further reduce costs through economies of scale and automation. Costs can be minimized further by replacing conventional polymer substrates with biodegradable cellulose-based materials, enhancing both affordability and sustainability. Furthermore, the smart packaging is compatible with existing high-speed methods, ensuring minimal cost impact.

**Table. S4|** Estimated cost of the smart packaging

| Component | Fabrication method | Materials | cost | Cost per unit | References |
| --- | --- | --- | --- | --- | --- |
| CNT gas sensor | Screen printing, spray-coating | Silver ink (LOCTITE® ECI 1011 E&C) | € 2556.66 per 1 Kg | € 0.2556 | ^[15]^ |
|  |  | CNT | € 225 per Kg | € 0.00025 | ^[16]^ |
| NFC antenna | roll-to-roll /screen printing | NFC | € 0.1 – 0.4 | € 0.4 | ^[17]^ |
| Active packaging | electrospinning | PPC polymer | € 317 euro per 100g | € 0.0317 | ^[18]^ |
|  |  | CEO oil | € 8.65 per 1 kg | € 0.05 | ^[19]^ |
|  |  | PEDOT:PSS (Clevios) | € 265 euro per 100 ml | € 0.1 | ^[20]^ |
| Encapsulation | Drop casting | PDMS | € 211 per 1 kg | € 0.211 | ^[21]^ |
| Total cost |  |  |  | € 1.048 | |

**References**

[1] K. M. Brodowska, A. J. Brodowska, K. Śmigielski, E. Łodyga-Chruścińska, **2016**, DOI 10.5281/ZENODO.197200.

[2] T. Rodrigues Arruda, P. Campos Bernardes, A. Robledo Fialho E Moraes, N. De Fátima Ferreira Soares, *Food Research International* **2022**, *156*, 111160.

[3] Z. Najmi, A. C. Scalia, E. De Giglio, S. Cometa, A. Cochis, A. Colasanto, M. Locatelli, J. D. Coisson, M. Iriti, L. Vallone, L. Rimondini, *Foods* **2023**, *12*, 332.

[4] A. Douaki, T. N. Tran, G. Suarato, L. Bertolacci, L. Petti, P. Lugli, E. L. Papadopoulou, A. Athanassiou, *Chemical Engineering Journal* **2022**, *445*, 136744.

[5] A. Dupas De Matos, E. Longo, D. Chiotti, U. Pedri, D. Eisenstecken, C. Sanoll, P. Robatscher, E. Boselli, *Foods* **2020**, *9*, 499.

[6] B. Moser, T. Steininger-Mairinger, Z. Jandric, A. Zitek, T. Scharl, S. Hann, C. Troyer, *Food Research International* **2023**, *172*, 113123.

[7] K. Broekaert, B. Noseda, M. Heyndrickx, G. Vlaemynck, F. Devlieghere, *International Journal of Food Microbiology* **2013**, *166*, 487.

[8] M. Mikš-Krajnik, Y.-J. Yoon, D. O. Ukuku, H.-G. Yuk, *Food Microbiology* **2016**, *53*, 182.

[9] A. B. Snyder, N. Martin, M. Wiedmann, *Nat Rev Microbiol* **2024**, DOI 10.1038/s41579-024-01037-x.

[10] O. A. Odeyemi, O. O. Alegbeleye, M. Strateva, D. Stratev, *Comp Rev Food Sci Food Safe* **2020**, *19*, 311.

[11] S. Xia, D. Fang, C. Shi, J. Wang, L. Lyu, W. Wu, T. Lu, Y. Song, Y. Guo, C. Huang, W. Li, *Food Chemistry* **2023**, *415*, 135752.

[12] L. Fu, Q. Xiao, Y. Ru, Q. Hong, H. Weng, Y. Zhang, J. Chen, A. Xiao, *International Journal of Biological Macromolecules* **2024**, *255*, 128196.

[13] A. Prasad, S. Khan, J. K. Monteiro, J. Li, F. Arshad, L. Ladouceur, L. Tian, A. Shakeri, C. D. M. Filipe, Y. Li, T. F. Didar, *Advanced Materials* **2023**, *35*, 2302641.

[14] P. Escobedo, M. Bhattacharjee, F. Nikbakhtnasrabadi, R. Dahiya, *IEEE Sensors J.* **2021**, *21*, 26406.

[15] “LOCTITE® ECI 1011 E&C, 511,33 €,” can be found under https://print-your-electronics-with-loctite.com/LOCTITEZ-ECI-1011-E-C, **n.d.**

[16] “Conductive Nanotubes Composite Additive - Cheap Tubes,” can be found under https://www.cheaptubes.com/product/conductive-nanotubes-composite/?add-to-cart=376, **n.d.**

[17] “Anti-falsi Auto Distruttivo A Prova Di Manomissione Etichette Nfc Antenna Chip Usa E Getta Rfid Tag Rfid Etichetta - Buy Rfid Tag,Tamerproof Rfid Tag,Anti-fake Rfid Tag Product on Alibaba.com,” can be found under https://italian.alibaba.com/product-detail/anti-fake-self-destructive-tamper-proof-60589408898.html, **n.d.**

[18] “Poly(propylene carbonate) average Mn 50,000 GPC 25511-85-7,” can be found under https://www.sigmaaldrich.com/IT/it/product/aldrich/389021, **n.d.**

[19] “Cinnamon Essential Oil,” can be found under https://www.indiamart.com/proddetail/cinnamon-essential-oil-2855837719848.html, **n.d.**

[20] “PH 1000 PEDOT:PSS,” can be found under https://www.ossila.com/products/pedot-pss-ph-1000, **n.d.**

[21] “SYLGARD 184, 1.1KG DOW, Kit elastomero, silicone bicomponente, fluido, Sylgard 184, temperatura ambiente, trasparente, 1,1kg | Farnell® Italia,” can be found under https://it.farnell.com/dowsil-formerly-dow-corning/sylgard-184-1-1kg/elastomer-clear-184-1-1kg/dp/101697, **n.d.**
